# Supplementary material for: The impact of national culture, altruism, and risk preference on salaries: The case of the Major League Baseball
Source: PLoS One. 2023 May 10;18(5):e0284556. doi: 10.1371/journal.pone.0284556 (PMC10171653; doi:10.1371/journal.pone.0284556)
Supplement: S1 Appendix — (DOCX) [file pone.0284556.s001.docx]

Appendix

Table A Literature correspondence

|  | Dependent Variable | Nationality/CD | | | Findings |
| --- | --- | --- | --- | --- | --- |
| This study | Logarithmic salary  (Logsal) | Pitcher | RE | NAT: 0.039 to 0.043  CD: 0.070 | 1. Nationality (NAT) and cultural distance (CD) are both significantly positively correlated with player salaries, indicating that salary discrimination exists in the MLB.  2. The risk preference and uncertainty avoidance of pitchers are significantly positively correlated with their salaries, and the collectivism and altruism of pitchers are significantly negatively correlated with their salaries; the collectivism and altruism of fielders are significantly positively correlated with their salaries.  3. The salary premium of players from Australia with the smallest CD from the USA (0.02) is 19.61% higher than that of players from South Korea with the largest CD from the USA (3.72). |
|  |  | Fielder | RE | NAT: 0.047 to 0.056  CD: 0.053 to 0.057 |  |
| Jane (2012) | Logarithmic salary  (logSal) | Fielder | RE | Race: 12349.38 to 16839.14 | 1. Inverse salary discrimination exists among the CPBL players from minority groups (i.e., indigenous players). While the other conditions are the same, indigenous players averagely earn a monthly salary of NTDs 25,000 to 27,000, 20% to 22% higher than the average salary of players. |
|  |  | Pitcher | RE | Race: 24516.44 to 28937.86 |  |
|  |  |  | 2sFE | Race: 32668.71 to 54732.82 |  |
| Jane, Chen and Kuo (2013) | Logarithmic salary (LogSal) | Fielder | OLS | NAT: 0.369 to 0.406 | 1. While other conditions remain unchanged, the average salary of international players is 54.7% to 57.3% higher than that of domestic players.  2. The QR results show that nationality has a significant impact on the salaries of high-salaried players, indicating that the salary premium of international players increases with their salaries. |
|  |  |  | QR | 25^th^ NAT: 0.287 to 0.350  50^th^ NAT: 0.328 to 0.436  75^th^ NAT: 0.394 to 0.508 |  |
|  |  | Pitcher | OLS | NAT: 0.219 to 0.246 |  |
|  |  |  | QR | 25^th^ NAT: 0.225 to 0.246  50^th^ NAT: 0.231 to 0.245  75^th^ NAT: 0.149 to 0.237 |  |
| Wang , Fang, and Wu (2017) | Player salary | Fielder | Nationality: 6,503 to 13,200 | | 1. For the professional baseball of South Korea, the salaries of foreign pitchers and fielders are higher than the salaries of domestic players, proving the existence of salary discrimination.  2. Consumer discrimination exists among the KBO players; specifically, baseball fans prefer to watch the participation of foreign players, and their attendance rate increases if foreign players participate in baseball games. |
|  |  | Pitcher | Nationality: 6,299 to 14,202 | |  |
| Jane (2021) | Logarithmic salary (LogSal) | Fielder | RE | Foreigner: 1.12 to 1.19  CD: 0.38 | 1. In Japan’s professional baseball, foreign fielders enjoy a salary premium of 0.93% to 0.95%, and foreign pitchers enjoy a salary premium of 0.74% to 0.76%.  2. The salary premium of players from Holland with the largest CD (6.44) is 10.45% higher than that of players from Italy with the smallest CD (0.94).  3. For professional baseball, fielders have different physiological advantages from pitchers. The BMI of fielders is significantly positively correlated with their salaries, whereas the BMI of pitchers is significantly negatively correlated with their salaries. |
|  |  |  | 2wFE | / |  |
|  |  |  | 2sFE | Foreigner: 0.93 to 0.95  CD: 0.31 |  |
|  |  | Pitcher | RE | Foreigner: 1.02  CD: 0.35 |  |
|  |  |  | 2wFE | / |  |
|  |  |  | 2sFE | Foreigner: 0.74 to 0.76  CD: 0.26 |  |
