# Supplementary material for: The impact of national culture, altruism, and risk preference on salaries: The case of the Major League Baseball
Source: PLoS One. 2023 May 10;18(5):e0284556. doi: 10.1371/journal.pone.0284556 (PMC10171653; doi:10.1371/journal.pone.0284556)
Supplement: S1 Text — (DOCX) [file pone.0284556.s002.docx]

**8 Endnotes**

^1^ Towers Perrin, a human resources and financial consulting firm, merged with Watson Wyatt in January 2010 to form Towers Watson, which provided reinsurance intermediary services and was active in actuarial consulting through its insurance consulting business (Source: Wikipedia).

^2^ The collectivism index indicates extreme collectivism if its value is 0, but indicates extreme individualism if its value is approximate to 100 (Hofstede, 2011).

^3^ Hypothesis 1: The larger the cultural distance with domestic players is, the greater the salary premium for the foreign player is.
