# Supplementary material for: The impact of national culture, altruism, and risk preference on salaries: The case of the Major League Baseball
Source: PLoS One. 2023 May 10;18(5):e0284556. doi: 10.1371/journal.pone.0284556 (PMC10171653; doi:10.1371/journal.pone.0284556)
Supplement: Attachment — Submitted filename: PLOS ONE.docx [file pone.0284556.s005.docx]

**The Impacts of National Culture, Altruism, and Risk Preference on Salaries: The Case**

**of the Major League Baseball**

**Overall comments**

The manuscript is publishable as it discusses an important research area. It also contains all the necessary components of a good research article. However, the authors should take note of the following:

- Update the literature review with more recent studies
- Carry out a comprehensive proofreading of the manuscript.
- Checking for the consistency of the citation with the references is also suggested.

Generally, to improve the quality of the manuscript, it is suggested for the authors to read a recent article on research methodology titled “Best practices in data collection and preparation: Recommendations for reviewers, editors, and authors” and integrate some of the recommendations of the authors of the article in their manuscript.

Thanks.
