# Supplementary material for: The impact of national culture, altruism, and risk preference on salaries: The case of the Major League Baseball
Source: PLoS One. 2023 May 10;18(5):e0284556. doi: 10.1371/journal.pone.0284556 (PMC10171653; doi:10.1371/journal.pone.0284556)
Supplement: Attachment — Submitted filename: 0330Response-to-Reviewers.docx [file pone.0284556.s007.docx]

PONE-D-22-24132R1

The Impacts of National Culture, Altruism, and Risk Preference on Salaries: The Case of the Major League Baseball

PLOS ONE

Dear Dr. Wang,

Thank you for submitting your manuscript to PLOS ONE. After careful consideration, we feel that it has merit but does not fully meet PLOS ONE’s publication criteria as it currently stands. Therefore, we invite you to submit a revised version of the manuscript that addresses the points raised during the review process.

Please submit your revised manuscript by Apr 30 2023 11:59PM. If you will need more time than this to complete your revisions, please reply to this message or contact the journal office at [plosone@plos.org](mailto:plosone@plos.org). When you're ready to submit your revision, log on to <https://www.editorialmanager.com/pone/> and select the 'Submissions Needing Revision' folder to locate your manuscript file.

Please include the following items when submitting your revised manuscript:

- A rebuttal letter that responds to each point raised by the academic editor and reviewer(s). You should upload this letter as a separate file labeled 'Response to Reviewers'.
- A marked-up copy of your manuscript that highlights changes made to the original version. You should upload this as a separate file labeled 'Revised Manuscript with Track Changes'.
- An unmarked version of your revised paper without tracked changes. You should upload this as a separate file labeled 'Manuscript'.

If you would like to make changes to your financial disclosure, please include your updated statement in your cover letter. Guidelines for resubmitting your figure files are available below the reviewer comments at the end of this letter.

If applicable, we recommend that you deposit your laboratory protocols in [protocols.io](http://protocols.io/) to enhance the reproducibility of your results. Protocols.io assigns your protocol its own identifier (DOI) so that it can be cited independently in the future. For instructions see: <https://journals.plos.org/plosone/s/submission-guidelines#loc-laboratory-protocols>. Additionally, PLOS ONE offers an option for publishing peer-reviewed Lab Protocol articles, which describe protocols hosted on [protocols.io](http://protocols.io/). Read more information on sharing protocols at <https://plos.org/protocols?utm_medium=editorial-email&utm_source=authorletters&utm_campaign=protocols>.

We look forward to receiving your revised manuscript.

Kind regards,

Muhammad Fareed, Ph.D
Academic Editor
PLOS ONE

Journal Requirements:

Please review your reference list to ensure that it is complete and correct. If you have cited papers that have been retracted, please include the rationale for doing so in the manuscript text, or remove these references and replace them with relevant current references. Any changes to the reference list should be mentioned in the rebuttal letter that accompanies your revised manuscript. If you need to cite a retracted article, indicate the article’s retracted status in the References list and also include a citation and full reference for the retraction notice.

**Response:**

We had updated the references on page 38 and listed as follows.

1. Jane, W. J. The impact of cultural distance on salary: the case of Samurai Japan. Eurasian Economic Review. 2021; 11: 85-123.
2. Asghar, F., & Asif, M. Salaries, performance and mediating effect of altruistic behavior: Fresh statistical evidence from the National Basketball Association. International Journal of Humanities, Art and Social Studies (IJHAS). 2018; 3: 71-9.
3. Pappas, J. M., & Flaherty, K. E. The moderating role of individual-difference variables in compensation research. Journal of Managerial Psychology. 2006; 21(1):19-35.
4. Williams, L.K. “Some correlates of risk taking”, Personnel Psychology. 1965; 18(3): 297‐310.

In table A of the Appendix on page 45, Jane (2020) is updated as Jane (2021).

**Additional Editor Comments:**

Reviewer#1

-> The paper was adjusted sufficiently in the text but figures should be reviewed. The quality of the plots is bad. Please, redo all figures to improve them.
**Response:**

We redo all figures to improve the quality of the plots. Thank you for the reminder.

Reviewer#2

->The discussion and implications section that should have been added before the conclusion section is still missing.
**Response:**

Thank you for the valuable suggestion. The discussion and implications are included in the text.

1. -On page 33, the discussion is rewritten as *“Many previous studies have demonstrated the race-related or nationality-related salary discrimination in professional sports [1,2,3,13,16,22], and their estimation results are compared with each other in Table A (see Appendix). Regarding the CD-related salary estimation in professional sports, Jane analyzed the salaries of Japan’s professional baseball players, finding that the salary premium of players from Holland with the largest CD (6.44) is 10.45% higher than that of players from Italy with the smallest CD (0.94) [5].*

*Moreover, other cross-cultural studies cover topics such as salary gap in multinational companies [24,25], impact of cultural background on financial and non-financial rewards [26], and professional players [5]. Many studies focused on salary gap caused by CD between company employees, but few studies have discussed the salary gap from the perspective of cross-cultural background in sports leagues in detail. In this study, we introduced related cultural indicators of players to examine salary gap caused by cultural differences between players and further estimate the salary premium of MLB players with different nationalities. As described in Table 7, players with a salary premium of not more than 19.73% are sequentially from South Korea, Panama, Japan, Saudi Arabia, and Venezuela. Players with the lowest salary premium (0.11%) are from Australia, followed by Canada, United Kingdom, South Africa, and Germany. The premium on salaries of Australian players who have the smallest CD (0.02) from the USA is 19.61% (=(3.72-0.02)*5.3%) higher than that of players from South Korea with the largest CD (3.72) from the USA.”*

2. -On page 35, implications are rewritten as *“The above analysis offsets the deficiency in studies that examined the salary discrimination in professional sports from a culture perspective. However, this study did not analyze the accurate salary gap between players with different nationalities, or estimate the salary gap using the QR method. In addition, this study merely introduced two cultural dimensions (including altruism and risk preference) of the six cultural dimensions in the GPS. Other cultural dimensions (e.g., power distance and trust) may help further explain the impact of cultural differences. Subsequent studies may verify the above conclusions and conduct an in-depth analysis considering the mutual effect of diverse complex factors. ”*

[Note: HTML markup is below. Please do not edit.]

Reviewers' comments:

Reviewer's Responses to Questions

**Comments to the Author**

1. If the authors have adequately addressed your comments raised in a previous round of review and you feel that this manuscript is now acceptable for publication, you may indicate that here to bypass the “Comments to the Author” section, enter your conflict of interest statement in the “Confidential to Editor” section, and submit your "Accept" recommendation.

Reviewer #1: (No Response)

Reviewer #3: All comments have been addressed

2. Is the manuscript technically sound, and do the data support the conclusions?

The manuscript must describe a technically sound piece of scientific research with data that supports the conclusions. Experiments must have been conducted rigorously, with appropriate controls, replication, and sample sizes. The conclusions must be drawn appropriately based on the data presented.

Reviewer #1: Yes

Reviewer #3: Yes

3. Has the statistical analysis been performed appropriately and rigorously?

Reviewer #1: Yes

Reviewer #3: Yes

4. Have the authors made all data underlying the findings in their manuscript fully available?

The [PLOS Data policy](http://www.plosone.org/static/policies.action#sharing) requires authors to make all data underlying the findings described in their manuscript fully available without restriction, with rare exception (please refer to the Data Availability Statement in the manuscript PDF file). The data should be provided as part of the manuscript or its supporting information, or deposited to a public repository. For example, in addition to summary statistics, the data points behind means, medians and variance measures should be available. If there are restrictions on publicly sharing data—e.g. participant privacy or use of data from a third party—those must be specified.

Reviewer #1: Yes

Reviewer #3: Yes

5. Is the manuscript presented in an intelligible fashion and written in standard English?

PLOS ONE does not copyedit accepted manuscripts, so the language in submitted articles must be clear, correct, and unambiguous. Any typographical or grammatical errors should be corrected at revision, so please note any specific errors here.

Reviewer #1: No

Reviewer #3: Yes

6. Review Comments to the Author

Please use the space provided to explain your answers to the questions above. You may also include additional comments for the author, including concerns about dual publication, research ethics, or publication ethics. (Please upload your review as an attachment if it exceeds 20,000 characters)

Reviewer #1: The discussion and implications section that should have been added before the conclusion section is still missing

Reviewer #3: The paper was adjusted sufficiently in the text but figures should be reviewed. The quality of the plots is bad. Please, redo all figures to improve them.

7. PLOS authors have the option to publish the peer review history of their article ([what does this mean?](https://journals.plos.org/plosone/s/editorial-and-peer-review-process#loc-peer-review-history)). If published, this will include your full peer review and any attached files.


If you choose “no”, your identity will remain anonymous but your review may still be made public.


**Do you want your identity to be public for this peer review?** For information about this choice, including consent withdrawal, please see our [Privacy Policy](https://www.plos.org/privacy-policy).

Reviewer #1: No

Reviewer #3: No

[NOTE: If reviewer comments were submitted as an attachment file, they will be attached to this email and accessible via the submission site. Please log into your account, locate the manuscript record, and check for the action link "View Attachments". If this link does not appear, there are no attachment files.]

While revising your submission, please upload your figure files to the Preflight Analysis and Conversion Engine (PACE) digital diagnostic tool, <https://pacev2.apexcovantage.com/>. PACE helps ensure that figures meet PLOS requirements. To use PACE, you must first register as a user. Registration is free. Then, login and navigate to the UPLOAD tab, where you will find detailed instructions on how to use the tool. If you encounter any issues or have any questions when using PACE, please email PLOS at [figures@plos.org](mailto:figures@plos.org). Please note that Supporting Information files do not need this step.
